# The Risk of Haematoma and Venous Thrombosis Associated With Thromboprophylaxis Use in Breast Cancer Surgery: A Meta-Analysis and Systematic Review

**DOI:** 10.1155/tbj/9898596

**Published:** 2025-02-05

**Authors:** Amenah Dhannoon, Ishwarya Balasubramanian, Ali A. Dhannoon, Abeeda Butt, Arnold D. K. Hill

**Affiliations:** ^1^Department of Breast Surgery, Royal College of Surgeons in Ireland, Dublin, Ireland; ^2^Department of Medicine, School of Medicine, University of Mosul, Mosul, Iraq

**Keywords:** anticoagulation, breast cancer surgery, breast conserving surgery, mastectomy, perioperative antithrombotic management

## Abstract

**Background:** The routine use of venous thromboembolism (VTE) prophylaxis in breast cancer surgery has caused substantial polarity among breast cancer surgeons across the globe. The aim of this study is to assess the use of VTE prophylaxis in breast cancer surgery outcomes.

**Methods:** A comprehensive electronic search was undertaken of all comparative studies that described the role of VTE prophylaxis in breast cancer surgery. Studies that reported on postoperative outcomes between patients who received VTE prophylaxis (prophylaxis) and those who did not (no prophylaxis) were included in the review. A meta-analysis using random-effect model was used to analyse key outcomes, with data presented as odd ratio (OR).

**Results:** A total of 2470 patients from 6 studies were included in this study. Among these patients, 60.9% (*n*: 1504) received prophylaxis. The haematoma rate in this study is 0.05% (*n*: 133). The incidence of haematoma was significantly associated with the use of prophylaxis (6.85% versus 3.11%, *p* : 0.001). Surgical intervention for haematomas was also significantly associated in this group (3.15% versus 0.83%, *p* : 0.004). However, there was no difference in VTE events between both groups (0.26% versus 0.36%, *p* : 0.88).

**Conclusions:** The use of VTE prophylaxis in breast cancer surgery is associated with increased haematomas without any benefit in preventing VTE events. Future studies that examine the use of risk assessment tools for VTE prophylaxis in high risk patients may be beneficial.

**Summary**

• There is no consensus on the use of prophylactic anticoagulant in breast cancer surgery.
• The use of prophylactic anticoagulant increases the risk of haematoma formation and risk of re-operation without any benefit in the reduction of thromboembolic events.
• Risk assessment tools specific to breast cancer surgery are required.

## 1. Introduction

The use of venous thromboembolism (VTE) prophylaxis has been well-documented in surgical oncology patients due to cancer associated thromboembolism [1, 2]. While there is substantial body of evidence to support the use of routine thromboprophylaxis in gastrointestinal and gynaecological patients [1–4], guidelines for VTE prophylaxis in breast cancer surgery are not well-established. This is largely due to concerns with the morbidity associated with postoperative breast haematoma and reoperations rates and its impact on length of stay, time to adjuvant therapy and oncological outcomes [5]. The rate of haemorrhagic events in breast cancer surgeries has been recorded as 0.4%–13% [6–11] with haematoma being the most common event (3.3%) as reported in a study of 540 patients by Andrea et al. [11]. Interestingly the American College of Chest Physicians recommends the use of VTE prophylaxis in breast cancer patients, as breast surgeries fall under “General Surgery” which also includes gastrointestinal surgery [12]. However, the American Society of Breast Surgeons (ASBrS) only advocates the routine use of VTE prophylaxis in high risk patients due to the relatively reported low incidence of VTE (0.16%) in breast procedures [13, 14]. Although VTE rate has been reported low in multiple large studies ranging from 0.2% to 0.75% [6, 8, 11, 14–16], The National Institute for Health and Care Excellence (NICE) does not provide any guidelines on VTE prophylaxis in breast cancer surgery patients [17] and the use of this in current practice is largely based on institutional experience.

Caprini score is a well-recognized tool in stratifying patients based on their individualized calculated risk for VTE from 40 different variables. A score of ≥ 5 would be an indication for the administration of VTE pharmacological prophylaxis [18]. In most breast cancer surgeries, two points are awarded for age of 60–75 years, one point for malignancy, two points for a procedure lasting more than 45 min [8]. While this qualifies majority of breast cancer surgery patients to fall under “high risk” for VTE events. Nevertheless, lower incidence of VTE events compared to what is predicted by the score has also been reported in one breast cancer center [8].

While a recent study by Klifto et al. compared the use of pharmacological and nonpharmacological VTE prophylaxis in breast surgery patients found that there is no significant difference between the two interventions in reducing VTE events or increasing risks of haematomas, the results were based on various breast procedures including surgical management of breast cancer, cosmetic, reconstructive, and/or prophylactic procedures and therefore there is insufficient evidence to determine whether VTE prophylaxis should be administered in breast cancer surgery patients [19].

This is the first study to look at VTE prophylaxis and its outcomes in breast cancer surgery exclusively. The main objectives of this study to assess whether breast cancer patients undergoing breast cancer should receive perioperative VTE prophylaxis or not through examining the rate of haematoma and VTE events associated with VTE prophylaxis administration in breast cancer surgery patients.

## 2. Materials and Methods

A systematic review and meta-analysis was performed according to the guidelines and recommendations from the preferred reporting items for systematic reviews and meta-analyses checklist (PRISMA) in Table A1 [20]. Institutional board and ethical committee approval were not required.

### 2.1. Literature Search and Study Selection

A systematic electronic search of PubMed, Embase, and Scopus was performed for all studies published looking at the role of prophylactic pharmacological VTE use and thromboembolic events in breast cancer surgery. Please find used search terms attached in Table A2.

The last search was performed on 14^th^ February 2024. Duplicated studies were manually removed. Two authors (A.D. and I.B.) examined the title and abstract of citations independently. Full texts of potentially eligible studies were obtained; disagreements were resolved by discussion with a third author (A.D.K.H.). The reference lists of retrieved papers were further screened for additional eligible publications. Lead authors of studies with incomplete or inadequate data were contacted by email for further information before being excluded from selected studies.

### 2.2. Eligibility Criteria

Comparative studies of the use of VTE prophylaxis and no VTE prophylaxis use with patients over the age of 18, who had breast cancer surgery [wide local excision (lumpectomy), simple or radical mastectomy with or without immediate breast reconstruction] and reporting data on the incidence of VTE events (deep venous thrombosis (DVT) and/or pulmonary embolism (PE)), and haematoma were eligible for inclusion. All studies reporting the use of VTE prophylaxis without comparative data were excluded. Studies that described cosmetic breast procedures, case reports, case series, editorials, reviews, and conference abstracts were excluded. There was no language restrictions applied in the initial search.

### 2.3. Data Extraction and Outcomes

Reviewers (A.D. and I.B.) recorded the following information independently regarding each eligible study: author's names, journal, year of publication, study type, enrolment dates, number of patients receiving VTE prophylaxis, and number of those who did not receiving VTE prophylaxis, mean age, surgical procedure and type of pharmacological agent administered. The following outcomes were recorded from included studies and used in the meta-analysis to compare outcomes in the settings of VTE prophylaxis administration and omission.

1. Primary outcome: haematoma rate.
2. Secondary outcomes: VTE events rate (DVT and PE), surgical intervention for haematoma.

### 2.4. Statistical Analysis

A random effects meta-analysis as described by DerSimonian and Laird was used to determine all pooled outcomes. The odds ratio (OR) was estimated using an inverse variance model and presented with 95% confidence intervals (CI) [21]. Heterogeneity in studies included was assessed by I-squared statistics and chi-square based Cochran's *Q* statistic test, in which *p* < 0.05 indicates the presence of significant heterogeneity. Low heterogeneity is considered in studies that *I*^2^ score 25–49 percent, moderate heterogeneity for *I*^2^ score 50–74% and over 75% for high degree heterogeneity [22]. Statistical analyses were conducted using Review Manager version 5 (The Nordic Cochrane Center, Copenhagen, Denmark).

### 2.5. Quality Assessment

**Figure 1:**
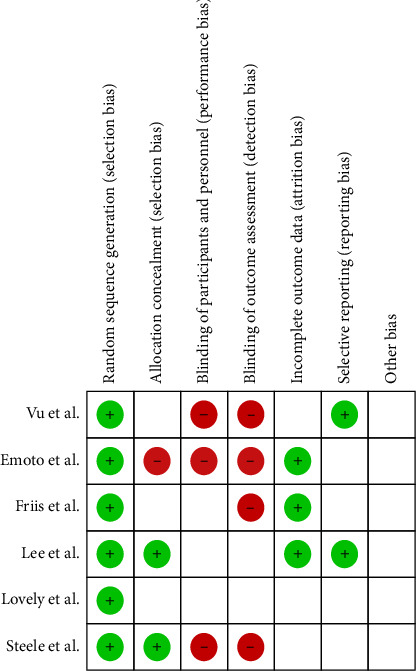
Evaluation of all potential bias in included studies with reference to Cochrane collaboration guidance. Green circle refers to low risk of bias and red circle refers to high risk of bias and unfilled squares refer to unclear risk.

The evaluation of the quality of included studies was performed according to the Cochrane handbook [23] (Figure 1). The risk of bias tools included selection bias, performance bias, detection bias, attribution bias, and reporting bias.

## 3. Results

### 3.1. Eligible Studies

**Figure 2:**
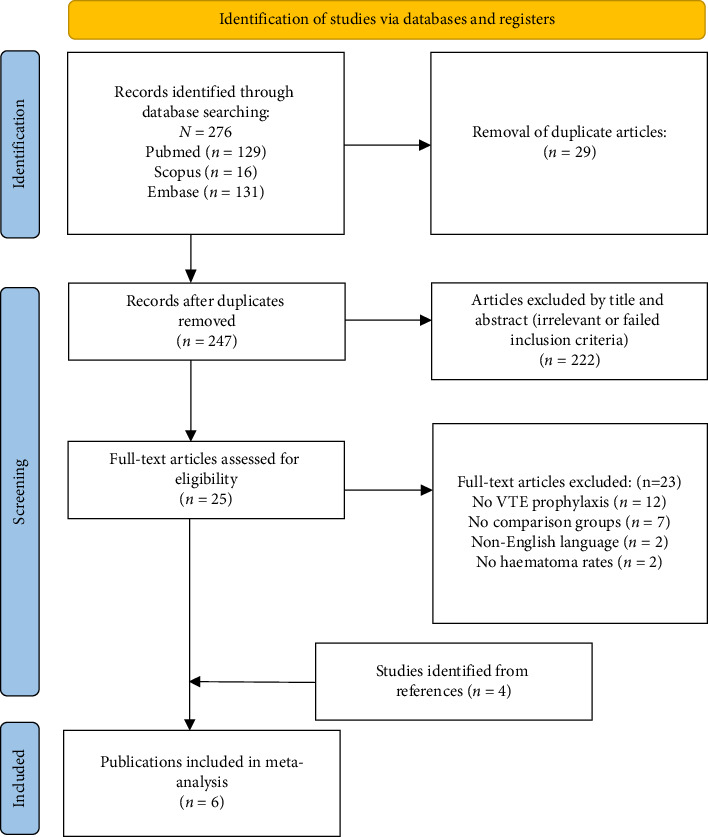
PRISMA diagram. Flow diagram of study selection.

Six studies containing comparative data on outcomes in patients who had breast cancer surgery in the prophylaxis and no prophylaxis groups were eligible for inclusion (Table 1) [8, 24–28]. The initial search identified 276 studies. A total of 29 duplicated studies were removed. Two hundreds and 47 studies were included in the title and abstract review. Twenty-five full text articles were assessed for eligibility, 23 of which were excluded. Four clinical studies were identified from references of two excluded studies, two of the studies [27, 28] were from a review paper by Patiar et al. [29] and the two studies [25, 26] were retrieved from the studies included in systematic review and meta-analyses by Klifto et al. [19]. Two of the studies were randomised controlled trials and four of the studies were performed retrospectively (Figure 2).

Mean age in all selected studies was 60.9 years. One-third of the studies were conducted in the USA [8, 24]. Of the remaining studies, two were conducted in UK [27, 28] one in Japan [25] and one study in Denmark [26]. All studies were conducted in a teaching hospital. A total of 2470 female patients were included. Of these patients, 60.9% (*n*: 1504) received prophylactic anticoagulant.

### 3.2. Incidence of Haematoma

**Figure 3:**
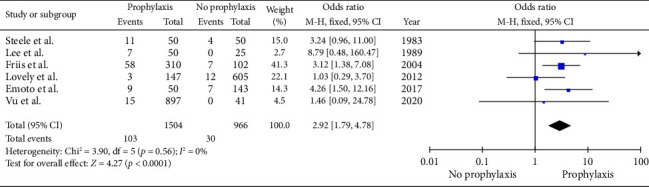
Meta-analysis of incidence of haematoma in breast cancer patients between the prophylaxis and the no prophylaxis groups. (*n*: 2470, *p* < 0.001, Cochrane *Q*: 3.90, df: 5, *p* < 0.56, I^2^: 0%).

The haematoma rate in this study is 0.05% (*n*: 133) across the six studies [8, 24–28]. There was a significant difference in the incidence of haematoma between the prophylaxis group (103/1504) versus the no prophylaxis group (30/966) (6.85% versus 3.11%, *p* : 0.001) (OD: 2.92: −0.65, 95% CI: 1.79–4.78) (Figure 3) without significant heterogenicity (Cochrane *Q*: 3.90, df: 5, *p* < 0.56, *I*^2^: 0%).

### 3.3. Incidence of Haematoma Requiring Surgical Intervention

**Figure 4:**
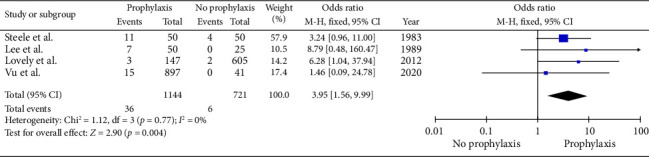
Meta-analysis of incidence haematoma requiring surgical intervention in the prophylaxis group versus the no prophylaxis group. (*n* = 1865, *p* : 0.004, Cochrane *Q*: 1.12, df: 3, *p* : 0.77, I^2^: 0%).

Four studies with 1865 patients described 42 patients requiring surgical intervention for postoperative haematoma [8, 24, 27, 28]. Surgical intervention for haematomas was also significantly associated in the VTE prophylaxis group versus the no prophylaxis group (3.15% versus 0.83%, *p* : 0.004) (OR: 3.95, 95% CI: 1.56–9.99, *p* : 0.004) (Figure 4). No significant heterogeneity was present between studies (Cochrane *Q*: 1.12, df: 3, *p* : 0.77, *I*^2^: 0%).

### 3.4. Incidence of VTE Events

**Figure 5:**
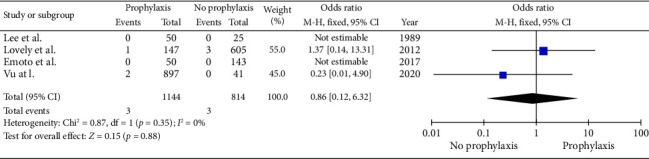
Meta-analysis of incidence of VTE events in the prophylaxis group versus the no prophylaxis group. (*n*: 1958, *p* : 0.88, Cochrane *Q*: 0.87, df: 1, *p* : 0.35, I^2^: 0%).

Four studies describing 1958 patients included data on the incidence of VTE events. However, there was no significant difference in VTE events between the prophylaxis and the no prophylaxis groups (0.26% versus 0.36%, *p* : 0.88) in the two studies that had VTE incidence. (OR: 0.86, 95% CI: 0.12–6.32, *p* : 0.35) (Figure 5). No significant heterogeneity was present between studies (Cochrane *Q*: 0.87, df: 1, *p* : 0.35, *I*^2^: 0%).

### 3.5. Breast Surgeries

All studies reported the use of prophylactic anticoagulant in simple mastectomy with radical mastectomy and breast conserving surgeries reported in 3 studies [8, 25, 26]. One study reported reconstruction in 159 of the patients who had mastectomies [8]. There was no breakdown of the types of reconstruction surgeries.

### 3.6. Type of Pharmacological Prophylaxis and Timings

The thromboprophylaxis included different pharmacological agents. This included subcutaneous low molecular weight heparin (LMWH) in two of the studies [8, 26] and unfractionated heparin (UFH) in all six studies making it the most anticoagulant used. With aspirin and warfarin cited in one of studies by Emoto et al. as this study used high thrombotic risk patients who are aspirin or warfarin users as the intervention arm of the study [25]. Additionally, timing for administration was not reported in all studies. In Lovely et al. perioperative administration was described referring to both preoperative and postoperative administration [8]. In Vu et al. 88.4% received preoperative heparin and 72.4% received postoperative as 5000 units of subcutaneous heparin every 12 h with 47.7% started evening of surgery and 52.3% the next morning. In earlier studies by Steele and Lee [27, 28], 5000 units of heparin was administered 2 h preoperatively and at 12 or 8, respectively, hourly until full mobilisation.

## 4. Discussion

There has been a substantial polarity among breast cancer surgeons regarding the use of perioperative thromboprophylaxis in breast cancer surgery patients [1, 12, 13, 29]. To our knowledge, this was the first systematic review and meta-analysis to assess the validity of pharmacological VTE prophylaxis in breast cancer surgery patients and weigh up the risks of haematoma and VTE events associated with the use of prophylactic anticoagulant in this population. Patients who received prophylactic anticoagulants were more likely to develop haematoma and to require surgical intervention than those who did not receive prophylactic anticoagulant. There was no difference in the rate of VTE events among those who received prophylactic anticoagulant and those who did not receive. There were no risk factors associated with the patients who developed these events. Pharmacological prophylaxis would not be recommended in the patients undergoing breast cancer surgery.

The studies that reported a comparison of two groups those who received pharmacological prophylactic anticoagulant and those who did not were 6 studies including two RCTs and 4 retrospective studies. The studies were performed in the UK, the USA, Europe, and Asia, creating generalisability to support our results. The use of funnel tests to assess heterogeneity was not possible due to the limited number of studies (6 studies). In addition, only two studies mentioned the timing of administration of the prophylaxis anticoagulant, there was a difference in the types of anticoagulant used in each study with UFH being most commonly used across all studies.

The haematoma rate in our study (0.05%) is similar to the published literature, and while the reported mean number of days for haematoma development is 4 days, the mean time for hematoma formation was not reported in any of the studies. According to Law et al. 21.4% of patients identified with haematomas were found to have an arterial bleed on return to theatre, which cannot be attributed to use of VTE prophylaxis [6]. On one hand, the increased risk of postoperative bleeding has also been associated with the use of other medications such as nonsteroidal anti-inflammatory drugs (NSAIDS) [30], there was no documented evidence on the use of such medication in our cohort except in one study by Friis that showed significant relationship between haematoma formation and the use of NSAID [26].

This review revealed some of the gaps in the published literature, such as omitting reporting of the important demographics associated with high risk VTE like the use of chemotherapy, body mass index (BMI), and other co-morbidities and thus limiting the ability to examine for confounding variables for high risk patients. Interestingly, all the VTE events occurred post mastectomy in Lovely et al.'s study were in nonsmoker, nonobese, younger than 60 patients without calculating Caprini score [8]. In contrast to Vu et al. who reported that the Caprini score for the two VTE incidences were 5 and 7 [24].

There are several limitations in this study. Four out of the six studies are retrospective cohort studies with data being collected from medical charts which may confer a risk for documentation error. Additionally, high risk of performance and detection biases is associated with administration of anticoagulants. While it would be optimal to have studies with randomised prospective collection of data including risk factors, use of prophylaxis and VTE and haematoma outcomes, we understand the financial and commitment challenges to this undertake, given the low rate of incidence of both of these outcomes in breast cancer surgery and the difficulty in demonstrating clinically relevant reduction (50% or greater).

Additionally, not all patients with thromboembolism are symptomatic and are incidentally picked up on imaging. To our knowledge, none of the studies screened asymptomatic patients. Another limitation is the studies included did not distinguish between high risk and low risk patients for VTE risk, with only one study describing the use of Caprini score in VTE incidence [24]. The included studies did not examine the effect of neoadjuvant chemotherapy, high BMI, and high tumour burden (T4 or nodal positivity) which are associated with high risk of VTE in breast cancer patients. Furthermore, in some studies only haematomas requiring return to theatre were tracked therefore, the number of nonoperative haematoma cannot be concluded. Additionally, with the increasing use of energy devices and improved surgical techniques [31], it is unclear if this has contributed to a reduction in the rate of haematoma reported across the studies. It is important to note that there was also no standardization in the type of surgical practice by the different surgeons across the different institutes in the 6 studies included.

## 5. Conclusion

The use of prophylaxis in breast cancer surgery is associated with increased haematoma formation and reoperation rates without any benefit in the prevention of thromboembolic events. Further studies that use specific risk assessment tools in identifying high risk patients will be useful in identifying patients that could benefit from VTE prophylaxis in breast cancer surgery.

## Figures and Tables

**Table 1:** Characteristics of included studies.

First author	Year	Country	Study type	Sample size	Time interval	VTE prophylaxis N	Non-VTE prophylaxis N
Vu et al. [24]	2020	USA	Retrospective	938	2012–2017	897	41
Emoto et al. [25]	2017	Japan	Retrospective	193	Jan 2010–Dec 2015	50	143
Lovely et al. [8]	2012	USA	Retrospective	752	Apr 2006–Jun 2010	147	605
Friis et al. [26]	2004	Denmark	RCT	412	Jun 1994–Aug 1996	310	102
Lee et al. [27]	1998	UK	RCT	75	—	50	25
Steele et al. [28]	1983	UK	Retrospective	100	Feb 1979–Jan 1980	50	50

**Table 2:** The preferred reporting items for systematic reviews and meta-analyses (PRISMA) guidelines, outlining the key criteria followed to ensure transparency and rigor in the review process.

Section and topic	Item #	Checklist item	Location where item is reported
*Title*			
Title	1	Identify the report as a systematic review	

*Abstract*			
Abstract	2	See the PRISMA 2020 for abstracts checklist	

*Introduction*			
Rationale	3	Describe the rationale for the review in the context of existing knowledge.	
Objectives	4	Provide an explicit statement of the objective(s) or question(s) the review addresses	

*Methods*			
Eligibility criteria	5	Specify the inclusion and exclusion criteria for the review and how studies were grouped for the syntheses	
Information sources	6	Specify all databases, registers, websites, organisations, reference lists and other sources searched or consulted to identify studies. Specify the date when each source was last searched or consulted	
Search strategy	7	Present the full search strategies for all databases, registers and websites, including any filters and limits used	
Selection process	8	Specify the methods used to decide whether a study met the inclusion criteria of the review, including how many reviewers screened each record and each report retrieved, whether they worked independently, and if applicable, details of automation tools used in the process	
Data collection process	9	Specify the methods used to collect data from reports, including how many reviewers collected data from each report, whether they worked independently, any processes for obtaining or confirming data from study investigators, and if applicable, details of automation tools used in the process	
Data items	10a	List and define all outcomes for which data were sought. Specify whether all results that were compatible with each outcome domain in each study were sought (e.g., for all measures, time points, analyses), and if not, the methods used to decide which results to collect	
	10b	List and define all other variables for which data were sought (e.g., participant and intervention characteristics, funding sources). Describe any assumptions made about any missing or unclear information	
Study risk of bias assessment	11	Specify the methods used to assess risk of bias in the included studies, including details of the tool(s) used, how many reviewers assessed each study and whether they worked independently, and if applicable, details of automation tools used in the process	
Effect measures	12	Specify for each outcome the effect measure(s) (e.g., risk ratio, mean difference) used in the synthesis or presentation of results	
Synthesis methods	13a	Describe the processes used to decide which studies were eligible for each synthesis (e.g., tabulating the study intervention characteristics and comparing against the planned groups for each synthesis (item #5))	
	13b	Describe any methods required to prepare the data for presentation or synthesis, such as handling of missing summary statistics, or data conversions	
	13c	Describe any methods used to tabulate or visually display results of individual studies and syntheses.	
	13d	Describe any methods used to synthesize results and provide a rationale for the choice(s). If meta-analysis was performed, describe the model(s), method(s) to identify the presence and extent of statistical heterogeneity, and software package(s) used	
	13e	Describe any methods used to explore possible causes of heterogeneity among study results (e.g. subgroup analysis, meta-regression)	
	13f	Describe any sensitivity analyses conducted to assess robustness of the synthesized results	
Reporting bias assessment	14	Describe any methods used to assess risk of bias due to missing results in a synthesis (arising from reporting biases)	
Certainty assessment	15	Describe any methods used to assess certainty (or confidence) in the body of evidence for an outcome	

*Results*			
Study selection	16a	Describe the results of the search and selection process, from the number of records identified in the search to the number of studies included in the review, ideally using a flow diagram	
	16b	Cite studies that might appear to meet the inclusion criteria, but which were excluded, and explain why they were excluded	
Study characteristics	17	Cite each included study and present its characteristics	
Risk of bias in studies	18	Present assessments of risk of bias for each included study	
Results of individual studies	19	For all outcomes, present, for each study: (a) summary statistics for each group (where appropriate) and (b) an effect estimate and its precision (e.g., confidence/credible interval), ideally using structured tables or plots	
Results of syntheses	20a	For each synthesis, briefly summarise the characteristics and risk of bias among contributing studies	
	20b	Present results of all statistical syntheses conducted. If meta-analysis was done, present for each the summary estimate and its precision (e.g. confidence/credible interval) and measures of statistical heterogeneity. If comparing groups, describe the direction of the effect	
	20c	Present results of all investigations of possible causes of heterogeneity among study results	
	20d	Present results of all sensitivity analyses conducted to assess the robustness of the synthesized results	
Reporting biases	21	Present assessments of risk of bias due to missing results (arising from reporting biases) for each synthesis assessed	
Certainty of evidence	22	Present assessments of certainty (or confidence) in the body of evidence for each outcome assessed	

*Discussion*			
Discussion	23a	Provide a general interpretation of the results in the context of other evidence	
	23b	Discuss any limitations of the evidence included in the review	
	23c	Discuss any limitations of the review processes used	
	23d	Discuss implications of the results for practice, policy, and future research	

*Other information*			
Registration and protocol	24a	Provide registration information for the review, including register name and registration number, or state that the review was not registered	
	24b	Indicate where the review protocol can be accessed, or state that a protocol was not prepared	
	24c	Describe and explain any amendments to information provided at registration or in the protocol	
Support	25	Describe sources of financial or nonfinancial support for the review, and the role of the funders or sponsors in the review	
Competing interests	26	Declare any competing interests of review authors	
Availability of data, code, and other materials	27	Report which of the following are publicly available and where they can be found: template data collection forms; data extracted from included studies; data used for all analyses; analytic code; any other materials used in the review	

*Note:* From: [20]. For more information, visit: https://www.prisma-statement.org/.

**Table 3:** The database search strategy, including the databases searched, keywords, Boolean operators, and filters applied to identify relevant literature. These tables support the reproducibility of the study by providing a clear framework for the literature selection process.

Embase	‘Breast tumor' OR ‘breast cancer' OR ‘breast disease' 1
	‘Breast surgery' OR ‘mastectomy' OR ‘partial mastectomy' OR ‘breast-conserving surgery' OR ‘subcutaneous mastectomy' OR ‘nipple-sparing mastectomy' 2
	(1 AND 2)-3
	‘Venous thromboembolism'/exp OR ‘venous thromboembolism' OR ‘vte' 4
	3 AND 4-5
	‘Chemoprevention' OR ‘anticoagulants' OR ‘anticoagulantion' OR ‘fibrinolytic agents' OR ‘chemoprophylaxis' OR ‘antifibrinolytic' OR ‘antifibrinolytics' 6
	((Venous AND thromboembolism AND prophylaxis OR vte) AND prophylaxis OR thromboembolic) AND prophylaxis OR ((anticoagulants OR anticoagulation) AND prophylaxis) OR ((low AND molecular AND weight AND heparin OR lmwh OR subcutaneous) AND heparin) OR enoxparin 7
	(6 OR 7)-8
	(5 AND 8)-

PubMed	“Venous thromboembolism” [MeSH terms] OR “VTE” [All fields] 1
	“Breast cancer” [All fields] OR “breast cancer surgery” [All fields] 2
	“Chemoprevention” [MeSH terms] OR “Anticoagulants” [MeSH terms] OR “Anticoagulants” [Pharmacological action] OR “fibrinolytic Agents” [MeSH terms] OR “fibrinolytic Agents” [Pharmacological action] OR “chemoprophylaxis” [Text word] OR “anticoagulant” [All fields] OR “Anticoagulants” [All fields] OR “antifibrinolytic” [All fields] OR “antifibrinolytics” [All fields] 3
	((“Venous thromboembolism” [MeSH terms] OR (“venous” [All fields] AND “thromboembolism” [All fields]) OR “venous thromboembolism” [All fields]) AND (“prevention and control” [MeSH subheading] OR (“prevention” [All fields] AND “control” [All fields]) OR “prevention and control” [All fields] OR “prophylaxis” [All fields] OR “prophylaxies” [All fields] OR “prophylaxy” [All fields])) OR (“VTE” [All fields] AND (“prevention and control” [MeSH subheading] OR (“prevention” [All fields] AND “control” [All fields]) OR “prevention and control” [All fields] OR “prophylaxis” [All fields] OR “prophylaxies” [All fields] OR “prophylaxy” [All fields])) OR ((“thromboembolic” [All fields] OR “thromboembolism” [MeSH terms] OR “thromboembolism” [All fields] OR “thromboembolisms” [All fields] OR “thromboembolization” [All fields]) AND (“prevention and control” [MeSH subheading] OR (“prevention” [All fields] AND “control” [All fields]) OR “prevention and control” [All fields] OR “prophylaxis” [All fields] OR “prophylaxies” [All fields] OR “prophylaxy” [All fields])) OR (“anticoagulants” [Pharmacological action] OR “anticoagulants” [MeSH terms] OR “anticoagulants” [All fields] OR “anticoagulant” [All fields] OR “anticoagulate” [All fields] OR “anticoagulated” [All fields] OR “anticoagulating” [All fields] OR “anticoagulation” [All fields] OR “anticoagulations” [All fields] OR “anticoagulative” [All fields] OR (“anticoagulants” [Pharmacological action] OR “anticoagulants” [MeSH terms] OR “anticoagulants” [All fields] OR “anticoagulant” [All fields] OR “anticoagulate” [All fields] OR “anticoagulated” [All fields] OR “anticoagulating” [All fields] OR “anticoagulation” [All fields] OR “anticoagulations” [All fields] OR “anticoagulative” [All fields]) OR ((“anticoagulants” [Pharmacological action] OR “anticoagulants” [MeSH terms] OR “anticoagulants” [All fields] OR “anticoagulant” [All fields] OR “anticoagulate” [All fields] OR “anticoagulated” [All fields] OR “anticoagulating” [All fields] OR “anticoagulation” [All fields] OR “anticoagulations” [All fields] OR “anticoagulative” [All fields]) AND (“prevention and control” [MeSH subheading] OR (“prevention” [All fields] AND “control” [All fields]) OR “prevention and control” [All fields] OR “prophylaxis” [All fields] OR “prophylaxies” [All fields] OR “prophylaxy” [All fields]))) OR (“heparin, low molecular weight” [MeSH terms] OR (“heparin” [All fields] AND “low molecular weight” [All fields]) OR “low-molecular-weight heparin” [All fields] OR (“low” [All fields] AND “molecular” [All fields] AND “weight” [All fields] AND “heparin” [All fields]) OR “low molecular weight heparin” [All fields] OR (“heparin, low molecular weight” [MeSH terms] OR (“heparin” [All fields] AND “low molecular weight” [All fields]) OR “low-molecular-weight heparin” [All fields] OR “lmwh” [All fields]) OR ((“subcutaneous” [All fields] OR “subcutaneously” [All fields] OR “subcutanous” [All fields]) AND (“heparin” [MeSH terms] OR “heparin” [All fields] OR “heparine” [All fields] OR “heparins” [All fields] OR “heparin s” [All fields] OR “heparinate” [All fields] OR “heparinated” [All fields] OR “heparines” [All fields] OR “heparinic” [All fields] OR “heparinisation” [All fields] OR “heparinised” [All fields] OR “heparinization” [All fields] OR “heparinize” [All fields] OR “heparinized” [All fields] OR “heparinizing” [All fields])) OR “enoxparin” [All fields]) 4
	1 AND 2 AND (3 OR 4)

SCOPUS	(TITLE-ABS-KEY (“venous thromboembolism” OR “VTE”)) AND (TITLE-ABS-KEY (‘breast AND tumor' OR ‘breast AND cancer' OR ‘breast AND disease')) AND (TITLE-ABS-KEY (‘breast AND surgery' OR ‘mastectomy' OR ‘partial AND mastectomy' OR ‘breast-conserving AND surgery' OR ‘subcutaneous AND mastectomy' OR ‘nipple-sparing AND mastectomy')) AND ((TITLE-ABS-KEY (‘chemoprevention' OR ‘anticoagulants' OR ‘anticoagulantion' OR ‘fibrinolytic AND agents' OR ‘chemoprophylaxis' OR ‘antifibrinolytic' OR ‘antifibrinolytics')) OR (TITLE-ABS-KEY (((venous AND thromboembolism AND prophylaxis OR vte) AND prophylaxis OR thromboembolic) AND prophylaxis OR ((anticoagulants OR anticoagulation) AND prophylaxis) OR ((low AND molecular AND weight AND heparin OR lmwh OR subcutaneous) AND heparin) OR enoxparin)))

## Data Availability

The data supporting this systematic review and meta-analysis are from previously reported studies and datasets, which have been cited. The processed data are available in the text and can be provided as a supporting file from the corresponding author upon request.

## Nomenclature

VTE: Venous thromboembolism
DVT: Deep venous thrombosis
PE: Pulmonary embolism
ASBrS: American Society of Breast Surgeons
NICE: National Institute for Health and Care Excellence
RCTs: Randomized controlled trials
PRISMA: Preferred reporting items for systematic reviews and meta-analyses
RR: Relative risk
CI: Confidence interval
SD: Standard deviation
